# Biological Maturation Predicts Dynamic Balance and Lower Limb Power in Young Football Players

**DOI:** 10.3390/biology11081167

**Published:** 2022-08-03

**Authors:** Bartosz Wilczyński, Łukasz Radzimiński, Agnieszka Sobierajska-Rek, Karol de Tillier, Jakub Bracha, Katarzyna Zorena

**Affiliations:** 1 Department of Immunobiology and Environment Microbiology, Medical University of Gdansk, 80-219 Gdansk, Poland; katarzyna.zorena@gumed.edu.pl; 2 Department of Physiology, Gdansk University of Physical Education and Sport, 80-336 Gdansk, Poland; lukaszradziminski@wp.pl; 3 Department of Rehabilitation Medicine, Faculty of Health Sciences with Institute of Marine and Tropical Medicine, Medical University of Gdansk, 80-219 Gdansk, Poland; agnieszka.sobierajska-rek@gumed.edu.pl; 4 Student Scientific Circle of Clinical Physiotherapy, Medical University of Gdansk, 80-219 Gdansk, Poland; karol.de.1998@gmail.com (K.d.T.); kubabracha5@gmail.com (J.B.)

**Keywords:** biological age, jump performance, young athletes, sport specialization, soccer

## Abstract

**Simple Summary:**

This study highlighted the importance of the relationship between dynamic balance and power of lower extremities in young male elite football players. Moreover, it revealed that biological status explains, to a large extent, jump performance and, to a lesser extent, dynamic balance in the studied group. The results of this study suggest that football academies, sport practitioners, and researchers should consider biological maturation when assessing athletic performance, designing rehabilitation, and strengthening training for sports injury analysis and prevention.

**Abstract:**

Biological maturation has an increasingly important role in youth sports. The aim of the study was to evaluate the relationship between dynamic balance and lower limb power and biological maturation in young football players. Seventy-two healthy, young male elite football players (age: 10 ± 2) participated in the study. Dynamic balance was assessed using a modified Star Excursion Balance Test (mSEBT). Power of the lower limbs was examined by a Countermovement Jump test (CMJ) and Single Leg Hop for Distance (SLHD). Furthermore, anthropometry and biological maturation (age, peak height velocity, and maturity offset) were assessed. There was a strong positive correlation between vertical jump (r = 0.75), distance long jump (r = 0.84), and biological maturation. A moderate positive correlation was found between dynamic balance (mSEBT COM, PL, and PM) and maturity offset. There was a significant association between mSEBT, CMJ, and SLHD (*p* < 0.05). Moreover, maturity offset explained 75% of vertical jump and 74% of distance long jump performance, respectively, and 12% of dynamic balance. Biological maturation should be considered when assessing athletic performance, establishing rehabilitation, and sports training in youth football players.

## 1. Introduction

In youth sports, there are positive and negative effects on the development of young athletes [1,2,3,4]. Benefits include improved overall health, self-confidence, and reduced risk of mental illness, among others [2,5,6]. On the other hand, the negative aspect is the association of practicing sports with the occurrence of injuries [4,7,8]. Numerous young athletes resign from a sport at the age of 15 (in the range of 14–17 years) [1,3]. The occurrence of an injury is considered one of the main reasons for this fact [9]. Moreover, the rate of injuries increases linearly (in the range from age 9 to 15) with a peak at age of 13 among young male football players [10,11]. The issue of injury in young football players is widely discussed and studied among medical professionals. Factors that may influence injuries are divided into external and internal. Internal factors include physical fitness and motor skills. There is evidence that strength/power in the lower extremities and dynamic balance limitations are among the internal risk factors for injury among children and adolescents [12,13].

Dynamic balance has been reported by numerous studies as having an association with lower extremity injury risk in various populations [14,15,16,17]. One of the well-studied, widely used, and non-instrumented tests for identifying balance deficits among athletes is the Star Excursion Balance Test (SEBT) [17]. The SEBT is a reliable and validated tool used to predict lower limb injuries and implement training programs in both patients and healthy participants [18]. For instance, poor performance on the SEBT has been a predictor of ankle sprains among recreational football players [19]. Moreover, previous ankle or knee injuries may predispose to reduced athletic performance, including reduced dynamic balance [20,21].

Lower extremity muscle strength is an essential component for maintaining and improving sports performance in young athletes. Muscle strength deficits in the lower extremity have been shown as a risk factor for injury among young adult football players [22] and youth basketball players [13]. To assess lower limb muscular power, measurements of maximal jumps (height and distance) are widely used by scientists and coaches. The Countermovement Jump (CMJ) test [23] is used for vertical assessment, while the Single Leg Hop for Distance (SLHD) assesses the jump length. There is evidence that poorer lower limb strength in the SLHD test may be associated with hamstring injury risk [24]. Furthermore, the scores of these two tests, CMJ and SLHD, respectively, in a recent study showed a significant, high correlation (r = 0.72) with each other among young rugby players, thus indicating that the variables are dependent on each other [8].

Furthermore, it was proven that there is a relationship between lower limb strength and dynamic balance in young athletes [8]. Other studies have shown that there were significant correlations between lower limb strength and balance among children [12].

Thus, a meaningful part of injury prevention in young sports may be the relationship between motor abilities and the biological maturation factor. Biological maturation refers to the progression into adulthood, which is individual, may depend on the biological system, and is categorized in terms of status, rate, and time [25,26]. A specific stage of maturation at the time of observation, such as skeletal age, is defined as maturity status [26]. Football players of the same chronological age may differ in maturity status by as much as 5–6 years in biological age [27]. Differences in biological maturity and chronological age can impact player selection and motor performance (strength, speed, balance) in youth football [27,28]. Such a phenomenon is called the Relative Age Effect (RAE). This may be due to the difference in experience and physical development that are associated with age. From around the age of 11, when the onset of puberty occurs, athletic performance, i.e., strength and power, also increases [28]. To calculate biological status, the formula by Mirwald [29] is commonly used. The formula has already been applied to youth alpine skiers [30,31] and ice hockey players [32]. By identifying, systematically evaluating, and educating about growth and maturation management and motor skills, practitioners can train young athletes more effectively [28].

The authors’ recent study revealed a significant association between jump performance and dynamic balance among youth rugby players [8]. From the standpoint of injury prevention (testing and improving athletic performance), it is important to know whether there is a relationship between dynamic balance and power of lower limbs and biological maturation in young athletes. Significant correlations between these variables may contribute to the scientific justification of injury risk assessment and the design of training programs, as well as rehabilitation. 

In the light of the research so far, the relationship between the described variables has not yet been studied in a population of young healthy football players. Therefore, our study aimed to evaluate the relationship between dynamic balance and lower limb power and biological maturation in a population of young football players. 

It was hypothesized that biological maturation will be significantly related to dynamic balance and lower limb power. Moreover, biological maturation could be considered an effective predictor of motor abilities in young male football players.

## 2. Materials and Methods

### 2.1. Study Design

This observational study was performed over the winter break (during the off-season period) from November 2021 to January 2022 and was completed on youth male elite football players at a rehabilitation and training center in a specially established research and sports gym. This study included: (i) the authors’ survey questionnaire and interview (inclusion and exclusion criteria), (ii) anthropometric and body composition analysis, and (iii) dynamic balance and lower limb power testing.

### 2.2. Participants

Inclusion criteria assumed that participants were in full health, pain-free, and had no surgical interventions in the lower extremities in the last 6 months. Signed consent from the parents/guardians of the young athletes was required to participate in the study prior to testing. Participants were excluded if they were unable to perform the test tasks or reported pain and if they participated in training and games irregularly.

### 2.3. Ethical Approval

This research was part of a scientific project (clinicaltrials.gov number: NCT04780880). The study was accepted by the Independent Bioethics Committee for Scientific Research in Gdańsk (approval number: NKBBN/680/2020) conforming to the recommendations of the Declaration of Helsinki.

### 2.4. Procedures

Before all tests, participants received information and education provided by the examiners regarding the conduct of the tests. The first testing phase consisted of taking a history (inclusion criteria, medical history, sports participation) and obtaining basic demographic and anthropometric data (age, standing height, sitting height). Baseline data (body weight, body mass index, muscle mass, percent of body fat) were obtained using a body composition analyzer (InBody 270, InBody Co., Seoul, Korea). Standing and sitting height and lower limb length were examined with a centimeter measure. Experienced researchers (B.W., K. de T., J.B.) in the next study phase administered bilateral (Countermovement Jump) and unilateral (Single Leg Hop for Distance) lower limb power tests and a dynamic balance assessment (Star Excursion Balance Test) to the participants. Data from the left lower extremities were used for statistical analyses. All investigators received additional theoretical and practical training in the conduct of the tests by the principal investigator (B.W.) at a rehabilitation and training center. Researchers received training over the course of one month (four full-day meetings), two months before the main measurement.

#### 2.4.1. Biological Status

Using data collected on the day of measurement (standing height, sitting height, body weight, and chronological age), biological status was calculated using the formula of Mirwald et al [29]. The method predicts the maturity offset (point in time, before or after reaching peak height velocity (PHV), of each participant). 

#### 2.4.2. Dynamic balance

The modified Star Excursion Balance Test (mSEBT) is a test designed for assessing dynamic balance and was originally invented as a rehabilitation exercise for the lower extremity. This test is commonly used for the evaluation of dynamic balance and postural control [17,33]. The participants were told to stand in the center of four intersecting lines forming a star. After that, participants were instructed to reach their foot as far as they were able to while keeping the whole surface of the standing foot on the ground. The participants were supposed to make 3 attempts in anterior (ANT), posteromedial (PM), and posterolateral (PL) directions for both the right and left lower limb. The test was deemed successful if participants were able to keep hands on their hips during the total movement and maintain a stable single-leg position. The length of each foot reach distance was measured and registered in centimeters. The result was presented as a % after being normalized to the length of the lower extremities. Lower limb length (LL) was assessed in supine position (anatomical points from the anterior superior iliac spine to the lateral malleolus). Composite (COM) distance was a value calculated by summing the distances of 3 directions and dividing it by 3 times the lower extremity length and multiplying by 100 [33]. Assessments of the SEBT reach distances have been demonstrated with high interrater reliability in previous studies [33].

#### 2.4.3. Vertical Jump

The Countermovement Jump (CMJ) was used to test the power of the lower extremities. The subjects were told to stand on a contact mat (Fusion Sport Smart Jump mat, Fusion Sport, 2 Henley ST, Coopers Plains, QLD, 4108, Australia) with both feet. The study participants were requested to keep their hands on their hips prior to and during the jump. The attempt was passed if the participant had straightened their knees during flight and initial contact. The participants took a 2 min break between jumps. Data analysis included the highest jump (cm) value from 3 attempts. CMJ allowed the assessment of the maximal vertical jump height while being a valid and reliable flight-time-based method [23,34,35].

#### 2.4.4. Distance Jump

Athletes were asked to put their hands on their hips while standing on one leg in front of the starting line. After the signal was given, the participant jumped as far as he could and landed on the same leg while keeping balance in one leg position for at least 2 s. The test was performed twice, one for each lower extremity with half a minute break between them. Jump length was measured and registered in centimeters. After 3 attempts the greatest distance for each leg was taken for analysis. SLHD is defined as the averaged distance of maximum jump for each leg marked as a dominant and non-dominant limb. In a recent study, the SLHD test exhibited excellent test–retest reliability [36].

### 2.5. Statistical Analysis

All variables had a normal distribution, which was examined by the Shapiro–Wilk test. Therefore, data were described as mean ± SD. Pearson correlation coefficient was used to analyze the relationships. Correlation strength (r) could be strong (0.50 ≤ r ≤ 1.0), moderate (0.3 ≤ r < 0.5), or weak (r < 0.3) [37]. Linear regression analysis was conducted to test the association and prediction of mSEBT, CMJ, and SLHD with maturity offset. The effect size of the linear regression was estimated with partial eta squared (*p*η^2^). The *p*η^2^ was classified as small (≥0.01), medium (≥0.06), and large (≥0.14) [38]. All data analyses were performed with the Statistica 13 (StatSoft, Krakow, Poland) software. The significance was previously established as *p* < 0.05.

## 3. Results

### 3.1. The Characteristics and Association between Calendar Age and Biological Status of The Studied Group of Young Football Players

**Figure 1:**
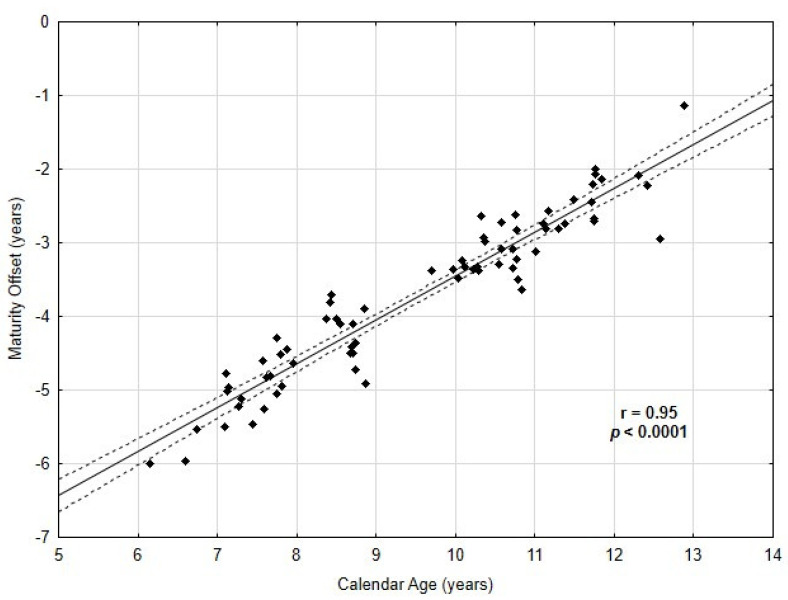
Correlations between Maturity Offset and Calendar Age.

Data were collected from 72 healthy, young male football players (age: 10 ± 2 years, maturity offset: −3.7 ± 1.1 years, peak height velocity: (13.3 ± 0.8 years), height: 139 ± 12 cm, body weight: 32 ± 7 kg) training at a professional football club in Poland. There was a strong significant correlation (*p* = 0.95, *p* < 0.0001) between calendar age and maturity offset in the studied group (Figure 1). The characteristics of the studied group of young football players are presented in Table 1. 

### 3.2. Relationship between Vertical Jump and Standing Long Jump

**Figure 2:**
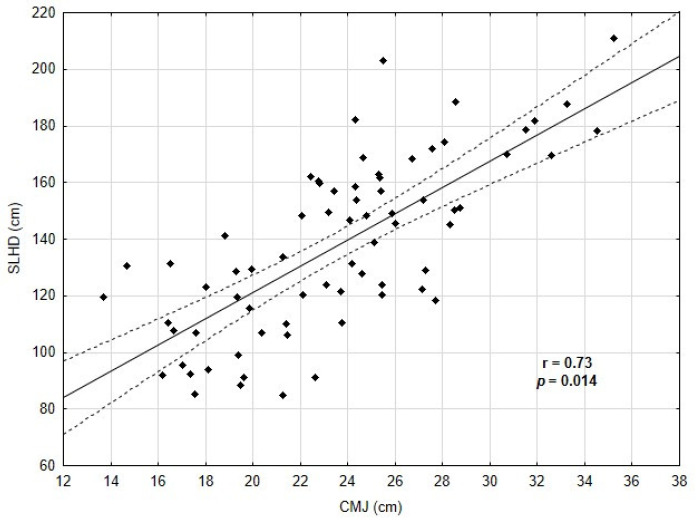
Correlations between CMJ and SLHD.

Data analysis revealed a strong positive correlation between CMJ and SLHD (r = 0.73, *p* = 0.014) among young football players (Figure 2).

### 3.3. Relationship between Dynamic Balance and Lower Limb Power

All dynamic balance scores except the PM direction had a relationship with lower limb power in the vertical jump (Table 2). A moderate positive correlation was observed between mSEBT COM (r = 0.38, *p* = 0.010) and mSEBT PL (r = 0.38, *p* = 0.010) and CMJ. A weak positive correlation occurred between mSEBT ANT (r = 0.29, *p* = 0.014) and CMJ. The mSEBT PM direction did not show a significant relationship with CMJ; however, the *p*-value was borderline (*p* = 0.052).

Dynamic balance and lower extremities power in the standing distance jump (SLHD) had significant weak correlations with mSEBT ANT direction (r = 0.25, *p* = 0.032) and moderate correlations with PL (r = 0.30, *p* = 0.012) and COM (r = 0.32, *p* = 0.006). There was no statistically significant (*p* = 0.073) correlation between mSEBT PM and SLHD.

### 3.4. Association between Dynamic Balance and Lower Limb Power with Biological Maturation

**Figure 3:**
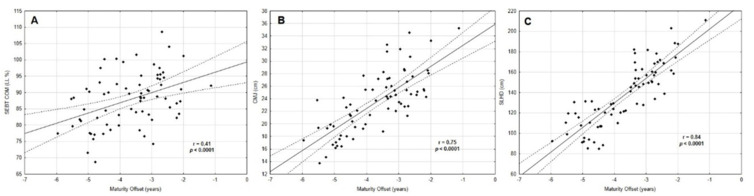
Correlations between (**A**) dynamic balance (**B**), (**C**) jump performance, and maturity offset.

Pearson correlation results assessing the association between mSEBT, CMJ and SLHD, and maturity offset are shown in Table 3. All dynamic balance scores except the ANT direction (*p* = 0.084) were associated with biological maturation. Moderate positive correlations occurred between mSEBT PL (r = 0.40, *p* = 0.001), PM (r = 0.33, *p* = 0.004), COM (r= 0.41, *p* < 0.0001), and maturity offset. Strong positive correlations (Figure 3) were visible between lower limb power, CMJ (r = 0.75, *p* < 0.0001), SLHD (r = 0.84, *p* < 0.0001), and maturity offset.

### 3.5. Dynamic Balance and Power of Lower Extremities as a Predictor for Maturity Offset 

The results of a linear regression model evaluating the association of mSEBT, CMJ, and SLHD with biological maturity in youth football players are shown in Table 4. Maturity shift explained 12% of the variance in mSEBT COMP with an effect size *p*η^2^ = 0.12, 75% in CMJ (*p*η^2^ = 0.56), and 73% in SLHD (*p*η^2^ = 0.73).

## 4. Discussion

Football players of the same chronological age can significantly differ in biological age, which can affect the assessment of their motor skills and team selection [27,28]. Research in football has led the way in disseminating the importance of biological maturation in youth sports [28]. We believe that our study has added its scientific contribution to this.

The main finding of this paper is that biological maturation status (maturity offset) was associated with dynamic balance and power of the lower extremities in young male elite football players. Maturity offset explained 75% of vertical jump and 74% of distance long jump performance, respectively, and to a lesser extent, only 12% of dynamic balance. Furthermore, CMJ and SLHD were directly strongly related to dynamic balance in the study cohort.

Our study found a strong positive correlation between vertical jump, distance long jump, and biological maturation in young male football players. The linear regression model showed a strong effect of maturity offset prediction on CMJ and SLHD scores. An explanation for the above results could be the thesis that neuromuscular adaptations influence the increase in muscle strength with biological maturity. Such conclusions were made by Gillen et al. [39], who highlighted this observation, especially in the context of studying large muscles and large strength differences that can be explained by biological maturity [39]. Moreover, mature children are significantly taller and heavier than their maturing colleagues (at age 9) [40]. Thus, more lean body mass assists in strength and power production [41].

Our results are in partial agreement with research by other authors [41,42]. For example, in the study of Itoh et al., where young elite football players participated, it was found that differences in biological maturity significantly affected muscle power. The authors concluded a practical implication in which coaches should carefully evaluate jump performance since these characteristics are affected by maturation status [43]. Moreover, a study by Almeida-Neto et al. found a strong relationship between lower extremity power (CMJ) and biological maturation (analyzed by PHV and bone age) in adolescent athletes (both sexes) [41]. On the other hand, Figueiredo et al. demonstrated no significant correlation between maturity and vertical jump performance among 11–12-year-old football players, but it was significant among 13–14-year-old football players [44].

The result of the moderate, positive correlation between maturity offset and mSEBT COM indicates that young athletes who were biologically older demonstrated greater dynamic balance. This is consistent with the widespread assumption that movement control may improve with age and experience in sports practice [45]. There is evidence that balance in children significantly increases with each month of development. This may result in major differences among same-aged children born in different quarters of the year [46].

The weak/moderate correlations and part of the nonsignificant correlations (mSEBT PM direction) between mSEBT and CMJ and SLHD in the presented study could suggest that adolescent athletes demonstrate an imbalance between strength and flexibility, which may lead to less coordination, also called "adolescent awkwardness" [47]. It should be added that our previous study found that, in a population of young rugby players, there were strong positive correlations between SLHD and Y-Balance Test and between CMJ and Y-Balance Test [8]. The difference between these results may be due to (1) the age of the population sample—the rugby players were significantly older than the football players—and (2) the different nature of the sport—rugby may require a greater engagement of muscular strength and power of the lower and upper extremities [7,8].

The strong association of jump performance tests (CMJ and SLHD) supports already existing findings in other populations of young athletes [8,48]. In a study on young rugby players, the same tests were used to assess lower extremity power. Thus, CMJ and SLHD also showed a strong positive correlation (0), similar to our results. A practical application would then be that both tests could be used interchangeably, as they are strongly dependent on each other.

## 5. Conclusions

This study highlighted the importance of the relationship between dynamic balance and power of the lower extremities in young elite football players. Moreover, it revealed that biological status may explain, to a large extent, jump performance and, to a lesser extent, dynamic balance in the studied group. The results of this study suggest that football academies, sport practitioners, and researchers should consider biological maturation when assessing athletic performance, designing rehabilitation, and strengthening training for sports injury analysis and prevention. However, the limitations of the study should be taken into account, and randomized trials should be performed in the future.

## 6. Study Limitations

Limitations of our study include the fact that we did not perform our test–retest reliability studies prior to our formal data collection. However, we relied on test–retest reliability assessments from other studies [23,33,35,36]. The study was conducted in a specific population sample of young male elite football players. Therefore, decisions about practical implications in other sporting populations should be made with caution, as evidenced by some of the differences in variable assessment discussed in this chapter using our previous study among older rugby players as an example. Furthermore, the nature of the study is based on examining these variables at a specific point in time. Future research should take this into account and attempt to follow changes in a reproducible manner over a long-term period of time.

## Figures and Tables

**Table 1:** Anthropometric, dynamic balance, and jump performance characteristics of participants.

Variable	N = 72	
	Mean	Std. Dev.
Age (years)	9.6	1.7
PHV (Peak Height Velocity) (years)	13.3	0.8
Maturity offset (years)	−3.7	1.1
Height (cm)	139.1	11.9
Body weight (kg)	32	7.3
BMI (Body Mass Index)	16.3	1.5
Percent body fat	14.7	4.1
Muscle mass	14.1	3.62
Dynamic balance—mSEBT		
Anterior LL%	70.5	7.7
Posterolateral LL%	93.4	10.6
Posteromedial LL%	100	12.9
Composite LL%	88	8.2
Jump Performance		
Countermovement Jump	23.6	4.8
Single Leg Hop for Distance	136.8	31.3

Abbreviation: mSEBT—modified Star Excursion Balance Test, LL %—Lower limb length in percent.

**Table 2:** Correlations between jump performance and dynamic balance.

		r	*p*-Value	r	*p*-Value
Jump Performance		Countermovement Jump		Single Leg Hop for Distance	
Dynamic Balance—SEBT	Anterior	0.29	0.014 *	0.25	0.032 *
	Posterolateral	0.38	0.01 *	0.30	0.012 *
	Posteromedial	0.23	0.052	0.21	0.073
	Composite	0.38	0.01 *	0.32	0.006 *

Abbreviations: mSEBT—modified Star Excursion Balance Test, r—Correlation coefficient, * statistically significant (*p* < 0.05).

**Table 3:** Correlations between jump performance, dynamic balance, and maturity offset.

		r	*p*-Value	Strength
Maturity offset (years)				
Dynamic Balance—mSEBT	Anterior	0.23	0.084	Weak
	Posterolateral	0.40	0.001 *	Moderate
	Posteromedial	0.33	0.004 *	Moderate
	Composite	0.41	<0.0001 *	Moderate
Jump Performance	Countermovement Jump	0.75	<0.0001 *	Strong
	Single Leg Hop for Distance	0.84	<0.0001 *	Strong

Abbreviations: mSEBT—modified Star Excursion Balance Test, r—Correlation coefficient, * statistically significant (*p* < 0.05).

**Table 4:** Linear regression model, between jump performance, dynamic balance, and maturity offset.

Predictor Variable	Dependent Variable	R²	*p*η^2^	β	SE ^b^	*p*-Value
Maturity offset	mSEBT Composite	0.12	0.12	0.353 (0.120, 0.576)	0.11	0.002
	Countermovement Jump	0.75	0.56	0.75 (0.587, 0.906)	0.08	<0.0001
	Single Leg Hop for Distance	0.73	0.73	0.85 (0.729, 0.978)	0.06	<0.0001

β—standardized regression coefficient, SE ^b^—Standard Error of standardized regression coefficients, CI—confidence intervals. Data are presented as regression coefficients *β* with standard error (SE **^b^**). Abbreviations: *p*η^2^—partial eta squared, mSEBT—modified Star Excursion Balance Test.

## Data Availability

The data presented in this study are available on request from the corresponding author.
